# Reply to Consavage Stanley, K.; Kraak, V.I. Comment on “Lau et al. Trends in Beef Intake in the United States: Analysis of the National Health and Nutrition Examination Survey, 2001–2018. *Nutrients* 2023, *15*, 2475”

**DOI:** 10.3390/nu15183936

**Published:** 2023-09-11

**Authors:** Clara S. Lau, Victor L. Fulgoni, Mary E. Van Elswyk, Shalene H. McNeill

**Affiliations:** 1 National Cattlemen’s Beef Association, a Contractor to the Beef Checkoff, 9110 East Nichols Ave., Suite 300, Centennial, CO 80112, USA; smcneill@beef.org; 2 Nutrition Impact, LLC, Battle Creek, MI 49014, USA; vic3rd@aol.com; 3 Van Elswyk Consulting, Inc., Clark, CO 80428, USA; mveconsulting@q.com

We thank the authors [1] for elevating attention on U.S. beef intake trends and appreciate the opportunity for further discussion. While more evidence on beef’s role in healthy, sustainable diets is critically needed, sustainability was outside the scope of our clearly defined research question which was determined before undertaking the analysis. The Beef Checkoff is one of twenty-two agriculture commodity promotion and research-oriented programs representing farmers, ranchers, and agricultural businesses that support the production of much of our nation’s food supply [2]. As a funder of scientific research, the Beef Checkoff is a stakeholder in protecting scientific integrity, and as scientists conducting research on behalf of the nation’s beef cattle farmers and ranchers, it is of paramount importance to us personally. Guiding principles for ethics in industry-funded research are well established and transparently available to the scientific community and public, and they have been followed to conduct and report this research, including fully disclosing information regarding conflicts of interest [3].

Because meat intake is often at the crux of scientific debate on optimal healthy, sustainable diets for the population [4], this research was conducted to provide descriptive beef intake data to compare to a relevant U.S. benchmark to help inform the public health community. More specifically, our research was simply an effort to provide objective data on beef intake trends using a publicly available database (i.e., National Health and Nutrition Examination Survey (NHANES)) in the context of the example patterns from the 2020–2025 Dietary Guidelines for Americans (DGA). We used methods commonly employed when evaluating food intake following NHANES analytical guidelines [5], and the use of NHANES allows for transparent and reproducible evidence. Usual intake is considered the most useful intake data to inform policy makers [6]. To our knowledge, we are the first to publish beef-specific usual intake data on both a per capita and consumer basis.

The DGA model dietary patterns that are intended to provide examples of healthy dietary patterns use food groups and subgroups rather than individual foods and beverages to avoid being prescriptive [7]. The modeling process underlying DGA sample dietary patterns is complex but is predicated on amounts and types of food consumed by Americans, meeting nutrient recommendations, and reducing the risk for chronic diseases. The amounts in the modeled DGA dietary patterns are intended to allow flexibility to choose a variety of foods and are not meant to imply the required intake of any particular food group/sub-group. In other words, as stated in our publication [8], a consumer may elect to consume beef as their protein of choice as part of a balanced diet that meets the goals of the DGA.

While the authors [1] compare the U.S. beef intake levels to EAT-Lancet Commission diet guidelines, it is important to note that the cost of the EAT-Lancet diet has been estimated to exceed household per capita income for at least 1.58 billion people and has been found to be nutritionally inadequate, particularly for populations with increased nutrient needs [9,10]. We recognize there are opportunities to improve human health and food systems through agricultural productivity, reduced food waste, decreased overconsumption of high-energy, low-nutrient foods, and emphasis on a healthy balance of nutrient-dense foods to ensure macro- and micro-nutrient needs are met within healthy dietary patterns. We look forward to continuing to advance and invest in rigorous scientific research that informs equitable food choice and maximizes the flexibility to choose healthy, sustainable dietary patterns that support each individual’s social, cultural, economic, and health needs.
